# Effectiveness of AI-Supported Game-Based Learning: A Systematic Review of Outcomes, Challenges, and Future Directions

**DOI:** 10.3390/bs16071050

**Published:** 2026-06-24

**Authors:** İsmail Kaşarcı, Eyüp Yurt

**Affiliations:** 1 Faculty of Education, Eskişehir Osmangazi University, 26040 Odunpazarı, Eskişehir, Türkiye; ikasarci@ogu.edu.tr; 2 Faculty of Education, Bursa Uludağ University, 16059 Nilüfer, Bursa, Türkiye

**Keywords:** artificial intelligence, game-based learning, serious games, large language models, dynamic difficulty adjustment, learning outcomes, motivation, systematic review

## Abstract

Background: AI-supported game-based learning (AI-GBL) integrates artificial intelligence mechanisms, including adaptive difficulty adjustment, large language model (LLM) scaffolding, intelligent non-player characters (NPCs), and stealth assessment, into game-based educational environments. Objective: This systematic review synthesizes the empirical evidence on AI-GBL effectiveness, adaptive mechanisms, and intelligent assessment approaches across diverse educational contexts. Method: Following PRISMA 2020 guidelines, 55 peer-reviewed empirical studies (2021–2026) were identified from Web of Science and Scopus databases. Two independent reviewers screened records (κ = 0.89; 100% consensus on disagreements), extracted data using a standardized coding scheme, and assessed methodological quality using a five-criterion rubric. A thematic synthesis approach was adopted due to the heterogeneity of the evidence base. Results: The reviewed studies generally suggest promising positive effects of AI-GBL on knowledge acquisition, intrinsic motivation, and affective engagement under a range of educational conditions. LLM-based scaffolding reduces cognitive load but risks fostering passive dependency; adaptive difficulty adjustment benefits depend critically on the direction and magnitude of adaptation; AI NPCs function as credible instructional partners in both EFL and STEM contexts; stealth assessment achieves AUCs of 0.848–0.913. Challenges include algorithmic bias in assessment models, LLM latency, over-reliance risks, and a near absence of longitudinal evidence. Conclusions: AI-GBL’s effectiveness rests on principled alignment between AI mechanisms and learning theory rather than algorithmic sophistication per se. Equity-by-design approaches and longitudinal evidence constitute the field’s priority research needs.

## 1. Introduction

Digital game-based learning (GBL) has emerged as one of the fastest-growing domains in educational technology research. Grounded in Vygotsky’s (1978) zone of proximal development, Mayer’s (2019) cognitive theory of multimedia learning, and Ryan and Deci’s (2000) self-determination theory, GBL leverages the intrinsic motivational properties of digital games to promote knowledge acquisition, skill development, and sustained engagement. Empirical syntheses have documented consistent positive effects across educational levels and subject domains (Mayer, 2019; Plass et al., 2020; Wouters et al., 2013), with the genre’s pedagogical value now recognized in formal education, vocational training, and clinical rehabilitation (Damaševičius et al., 2023; Mitsea et al., 2025).

Concurrently, artificial intelligence has transformed scale, accessibility, and educational applicability (Bozer Özsaraç & Ergin, 2025). The successive emergence of machine learning, deep learning, reinforcement learning, and large language models (LLMs) has created an expanding repertoire of techniques capable of personalizing learning, adapting instructional difficulty in real time, generating contextually rich feedback, and modeling complex learner states (X. Chen et al., 2022; Porayska-Pomsta et al., 2024; UNESCO, 2023). These capabilities are particularly consequential for game-based learning environments, where static, scripted game behaviors have historically constrained the individualization and responsiveness that effective pedagogy demands (Streicher & Smeddinck, 2016; Westera et al., 2020).

The integration of AI within game-based learning, hereafter referred to as AI-supported game-based learning (AI-GBL), represents a qualitatively distinct paradigm. AI-GBL environments simultaneously harness game design’s motivational affordances (challenge, narrative, feedback loops, flow states) and AI’s adaptive, data-driven capabilities (learner modeling, stealth assessment, dynamic difficulty adjustment, intelligent non-player characters). This convergence enables immersive, personalized, and continuously assessed experiences without the disruptions of conventional testing (Rahimi & Shute, 2024; Shute & Ke, 2012).

The existing review literature on AI-GBL has notable limitations. X. Tao et al. (2025) conducted an enhanced meta-review of AI applications in serious games within the health sector, providing a comprehensive, but domain-restricted, contribution. Mitsea et al. (2025) examined serious games assisted by AI, immersive technologies, the metaverse, and neurotechnologies for meta-skills training, offering a theoretically rich account but heterogeneous in its technology scope and not primarily focused on AI mechanisms as a unit of analysis. Neither review addresses the full range of educational contexts, AI mechanism types, and outcome dimensions characterizing the contemporary AI-GBL landscape.

The present systematic review addresses this gap. The review synthesizes 55 peer-reviewed empirical studies (2021–2026) identified through Web of Science and Scopus, following PRISMA 2020 guidelines, and is guided by three research questions:

RQ1: What are the documented effects of AI-supported game-based learning on learners’ academic outcomes, motivational states, and affective experiences?

RQ2: What AI mechanisms and adaptive personalization strategies are employed in game-based learning environments, and how do they function to support individual learner differences?

RQ3: What intelligent assessment approaches are used within AI-GBL environments, and what are the principal challenges and future research directions?

By addressing these questions, the present review aims to offer actionable insights for multiple stakeholder groups. For instructional designers and game developers, the synthesized evidence on adaptive mechanisms and intelligent NPCs provides empirically grounded design principles. For educators and policymakers, the review clarifies the conditions under which AI-GBL yields meaningful learning gains. For researchers, the identified methodological gaps, particularly the scarcity of longitudinal and equity-focused studies, delineate a concrete agenda for future investigation.

## 2. Theoretical Background

The theoretical foundation of AI-GBL draws on three converging bodies of scholarship. From educational psychology, cognitive load theory (Sweller, 2011) explains why well-designed adaptive mechanisms improve learning. By calibrating task difficulty to individual working memory capacity, AI reduces extraneous cognitive load and frees resources for germane processing. In the context of the present review, this framework serves as a primary lens for evaluating how AI-driven adaptive difficulty adjustment and LLM-based scaffolding modulate the cognitive demands placed on learners (RQ2).

Self-determination theory (Ryan & Deci, 2000) accounts for motivational dynamics: effective AI-GBL supports competence (through appropriate challenge calibration), autonomy (through responsive feedback and learner agency), and relatedness (through AI characters simulating social presence). This theory underpins our analysis of motivational and affective outcomes across the reviewed studies, particularly regarding the extent to which different AI mechanisms differentially satisfy these three basic psychological needs (RQ1).

Vygotsky’s (1978) zone of proximal development provides the scaffolding rationale: LLM-based tutors and adaptive NPCs can position instructional support within the learner’s developmental range in ways that static game content cannot. The ZPD framework is especially pertinent to our examination of LLM-powered scaffolding and intelligent NPC interactions, where the central design challenge is calibrating support that is neither too directive nor too minimal (RQ2).

From the game-based learning literature, the flow theory of optimal experience (Csikszentmihalyi, 1990) is foundational: games sustain engagement through the balance of challenge and skill, and AI-driven adaptive difficulty directly operationalizes this balance in real time. Shute and Ke’s (2012) evidence-centered design framework for stealth assessment establishes the psychometric basis for measuring learner competencies through gameplay behaviors, while Plass et al.’s (2020) cognitive-affective-motivational model of game-based learning situates GBL’s effectiveness within the interplay of cognitive processing, emotional engagement, and motivational regulation; all three of which AI mechanisms can address simultaneously.

Together, these frameworks position AI-GBL not as a technological enhancement of games, but as a theoretically coherent instructional paradigm in its own right. Accordingly, the present review uses these theoretical foundations not merely as background context but as evaluative criteria against which the empirical evidence is systematically appraised.

## 3. Method

This study employed a systematic review design following the PRISMA 2020 guidelines (Page et al., 2021). The completed PRISMA 2020 Checklist is provided in the Supplementary Materials. The overarching research question addressed the effectiveness of AI mechanisms integrated within game-based learning environments across educational contexts, motivational dimensions, and assessment approaches. The review protocol was retrospectively registered on the Open Science Framework (OSF); registration available at: https://osf.io/2a8jp (accessed on 25 May 2026).

### 3.1. Search Strategy

A systematic literature search was conducted on 20 May 2026 across Web of Science (WoS) Core Collection and Scopus. These databases were selected for their complementary indexing scope and demonstrated suitability for systematic reviews in educational technology and computer science (Gusenbauer & Haddaway, 2020). The WoS Topic (TS) field query combined four Boolean strands covering AI terms (artificial intelligence, machine learning, deep learning, generative AI, large language model*, LLM*, ChatGPT, intelligent tutoring system*, adaptive learning), GBL terms (game-based learning, digital game-based learning, serious game*, educational game*), outcome terms (learning outcome*, academic achievement, performance, engagement, motivation), and population terms (student*, learner*, school*, K-12, higher education). This returned 477 records; post-search filters (last five years; Article/Early Access; English; relevant subject categories) yielded 140 records. The Scopus TITLE-ABS-KEY query used analogous terms, returning 829 records; filters (last five years; English; journals; Social Sciences, Computer Science, Psychology) yielded 169 records.

The “Article/Early Access” document-type filter was activated in both databases, ensuring that online-first and in-press articles formally indexed prior to 20 May 2026 were captured. All 2026 publications included in the final sample had received a DOI and been formally indexed at the time of retrieval; full bibliographic details are provided in Appendix A (Table A1) to ensure complete replicability of the search protocol.

### 3.2. Study Selection

Following deduplication (59 cross-database duplicates removed by DOI and title matching), 250 unique records were screened independently by two authors against pre-specified inclusion/exclusion criteria (Table 1). Inclusion required an empirical design, AI explicitly integrated into a GBL environment, at least one measurable learning-related outcome, an educational context, and a peer-reviewed English journal publication within the last five years. Review articles, conceptual papers, gamification-only studies without AI integration, and entertainment games without educational intent were excluded. Initial inter-rater agreement was 92% (Cohen’s κ = 0.89); all 20 discrepant records were resolved through structured discussion, yielding 100% consensus. The corrected and internally consistent PRISMA flow is: WoS (n = 140) + Scopus (n = 169) = 309 identified → 59 duplicates removed → 250 screened → 153 excluded at title/abstract stage → 97 assessed for full-text eligibility → 42 excluded at full-text stage → 55 studies included.

The 42 full-text exclusions comprised: 17 studies in which AI was not involved in the learning process itself (EC6); 8 studies that did not measure a learning outcome (IC5); 5 studies that were not empirical in nature (IC2); 4 studies that lacked an educational context (IC4); 4 studies focused on gamification only without AI-GBL integration (EC4); and 4 studies that were not published as peer-reviewed journal articles (IC6). A systematic re-audit of all retained studies against the four core inclusion criteria was conducted; results are reported in the IC Compliance column of Table A1 (Appendix A).

**Figure 1:**
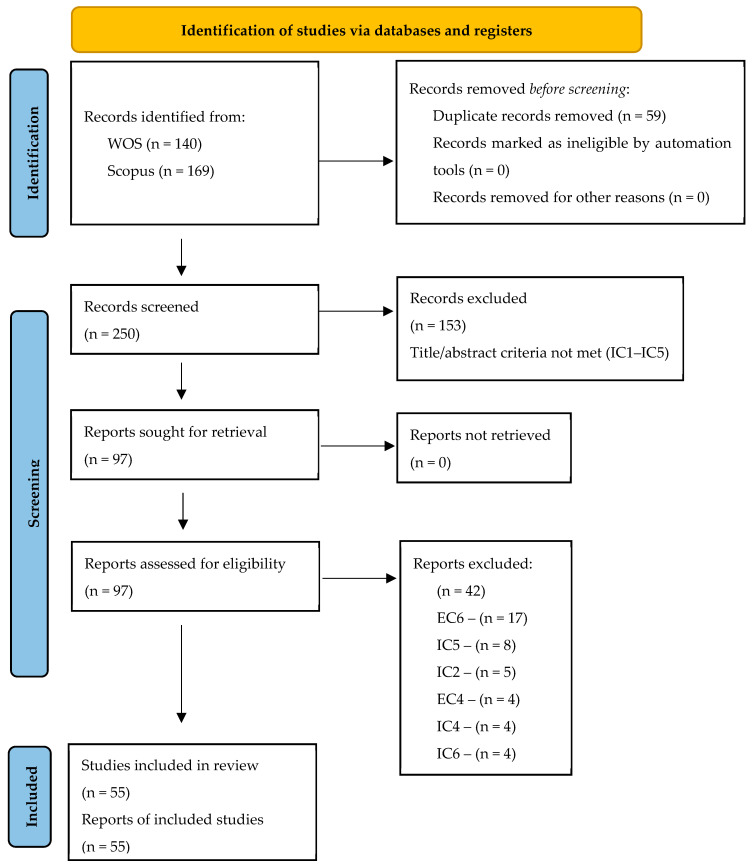
PRISMA 2020 Flow Diagram Illustrating the Study Selection Process.

Figure 1 presents the PRISMA 2020 flow diagram documenting all stages of the study selection process, from initial database identification through deduplication, title/abstract screening, full-text eligibility assessment, and final inclusion (n = 55).

### 3.3. Data Extraction and Quality Assessment

Data extraction was conducted independently by both authors using a standardised coding scheme capturing eight categories: bibliographic information, study design, AI mechanism type and role, GBL format and domain, outcomes measured, assessment instruments, key findings and effect sizes, and quality indicators (Table 2). Disagreements were resolved through discussion.

Methodological quality was assessed using a five-criterion rubric (Table 3), each criterion scored 1–5, yielding a total of 5–25. Quality tiers: High (≥21), Moderate (16–20), Low (≤15). Both authors independently scored all studies; discrepancies were resolved through discussion. The rubric was developed inductively, drawing on criteria established in prior systematic reviews and meta-analyses in educational technology and game-based learning (Mayer, 2019; Wouters et al., 2013), and adapted to address methodological demands specific to AI-GBL studies. The five criteria: research design rigor, sample size, outcome measurement validity, AI transparency, and generalisability, were selected to capture dimensions of internal validity, external validity, and replicability that are particularly salient in heterogeneous AI-GBL research contexts. Specific thresholds (e.g., n ≥ 200 for maximum sample size score) were anchored to benchmarks commonly reported in quantitative synthesis of educational technology interventions (Wouters et al., 2013). Although the rubric has not been externally validated (a limitation we explicitly acknowledge), inter-rater reliability was strong (Cohen’s κ = 0.89), supporting its consistent application.

An important AI mechanism taxonomy was developed inductively to distinguish five functional categories of AI role in included studies: (1) AI integrated during gameplay for real-time adaptation (DDA, intelligent NPCs); (2) AI for stealth assessment and performance analytics; (3) AI for scaffolding and feedback (LLM-based tutors); (4) AI for post-hoc learning analytics; (5) AI as external support without direct GBL integration. This taxonomy is applied consistently in the synthesis sections and in the IC Compliance column of Table A1.

### 3.4. Data Synthesis

Given the methodological heterogeneity of the included studies, a quantitative meta-analysis was not feasible for four reasons. First, effect sizes were inconsistently reported or absent across a substantial proportion of the included studies, precluding a common metric for aggregation. Second, outcome measures varied considerably across studies, spanning validated psychometric instruments, bespoke pre/post-content tests, physiological sensors, log-based behavioral indicators, and qualitative ratings, making direct comparison analytically inappropriate. Third, the AI mechanisms under examination (ranging from rule-based DDA to LLM scaffolding to deep learning stealth assessment) represent conceptually distinct intervention types that cannot be meaningfully collapsed into a single treatment category. Fourth, comparison conditions differed widely, including no-intervention controls, conventional instruction, non-AI game-based learning, and other AI tools, further compounding incompatibility.

Thematic synthesis (Thomas & Harden, 2008) was therefore employed as the most appropriate method for this evidence base, involving line-by-line coding, the development of descriptive themes, and the generation of analytical themes that extend beyond individual studies. Five thematic clusters emerged: learning outcomes, motivational and affective dimensions, AI-driven personalization and adaptation, intelligent assessment and analytics, and challenges and future directions.

### 3.5. Descriptive Overview of Included Studies

**Figure 2:**
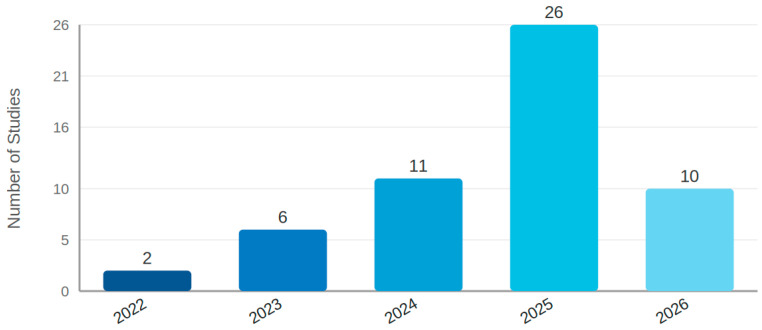
Distribution of Included Studies by Publication Year (n = 55).

**Figure 3:**
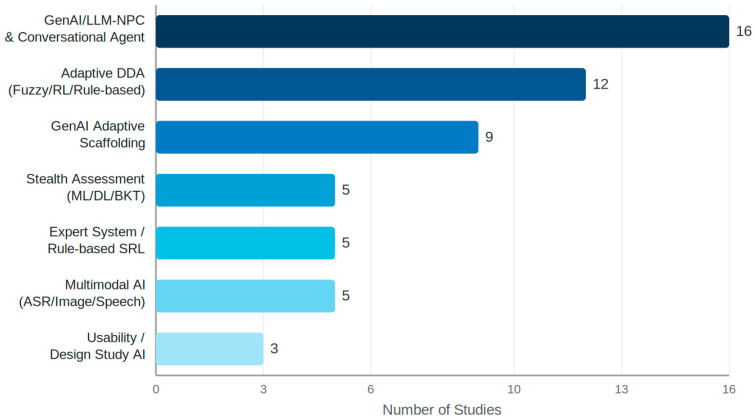
AI Mechanisms Employed Across Included Studies (n = 55).

**Figure 4:**
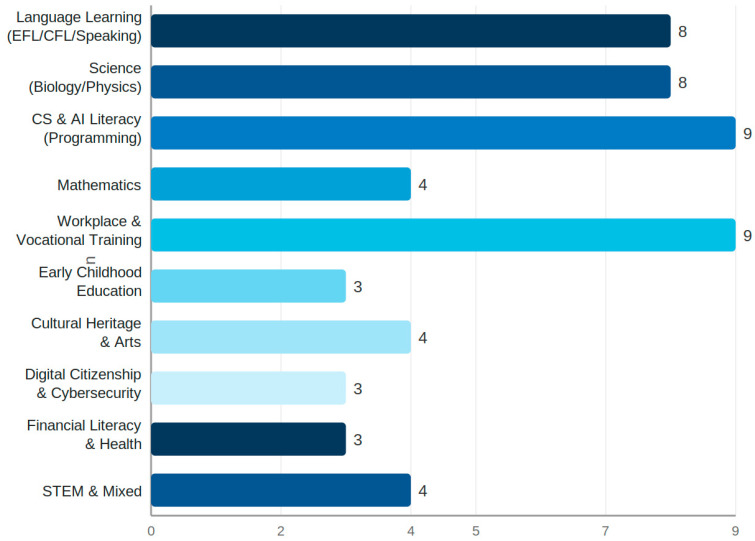
Distribution of Included Studies by Subject Domain (n = 55).

**Figure 5:**
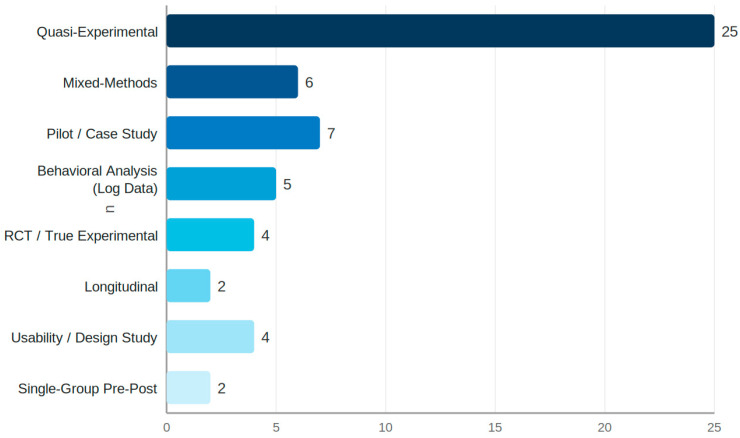
Distribution of Included Studies by Research Design (n = 55).

**Figure 6:**
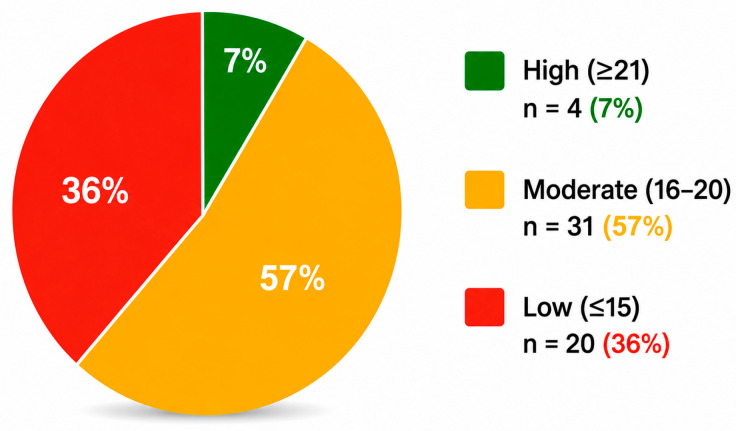
Distribution of Methodological Quality Ratings (n = 55).

**Figure 7:**
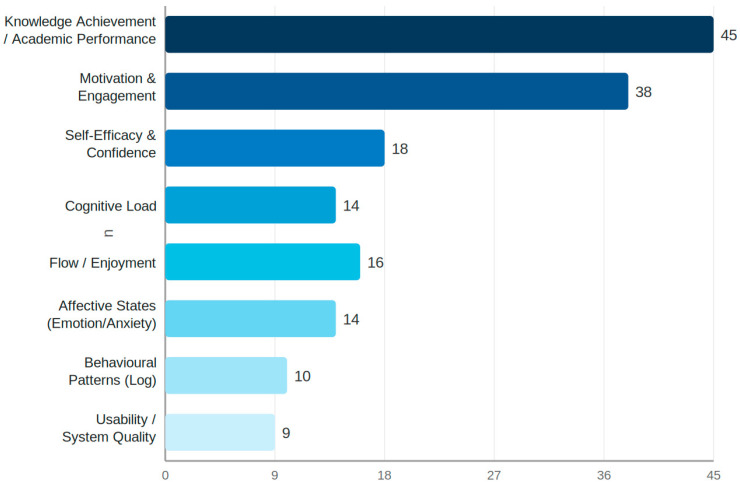
Frequency of Reported Outcome Categories Across Included Studies (Studies may report multiple outcomes).

Figure 2, Figure 3, Figure 4, Figure 5, Figure 6 and Figure 7 summarise the characteristics of the 55 included studies. Publication frequency increased markedly across the review period (Figure 2), with the highest volume in 2025 (n = 26, 47%), reflecting the rapid growth of LLM and generative AI applications in educational gaming following the widespread availability of these technologies from late 2023 onwards. LLM/generative AI-based mechanisms (n = 24, 44%) and adaptive difficulty adjustment (n = 15, 27%) were the most frequently employed AI mechanism categories (Figure 3). Language learning and EFL/CFL contexts (n = 8, 15%) and science education (n = 8, 15%) were the most common subject domains (Figure 4). Quasi-experimental designs with control groups predominated (n = 25, 45%), followed by mixed-methods and behavioural analysis studies (Figure 5). Quality assessment revealed that 7% of studies were rated High quality, 57% Moderate, and 36% Low (Figure 6; mean score = 16.1, SD = 2.9, range = 9–21).

## 4. Findings

The findings are organised into five thematic clusters. An overview of all 55 included studies is provided in Appendix A (Table A1); methodological quality ratings are provided in Appendix B (Table A2). Thematic summary tables are presented within each major section below.

### 4.1. Learning Outcomes and Academic Performance

#### 4.1.1. Knowledge Acquisition and Skill Development

AI-supported game-based learning environments showed positive learning effects across diverse subject domains in the majority of included studies. However, effect sizes and statistical significance varied considerably by study design quality and outcome measure. In mathematics, Kulkarni et al. (2025) reported a 6.03-point gain in scores following an AI-powered arithmetic game for primary children. Liu and Ldokova (2026) demonstrated large, sustained mathematical achievement gains in an RCT (n = 120) comparing GPT-powered games against standard online instruction. Santos et al. (2026) documented a 58% increase in financial performance and a 37% reduction in maladaptive gambling behaviors through a multi-agent generative AI financial literacy game (n = 134; children aged 7–12).

In science, C.-H. Chen and Chang (2024) found that ChatGPT-assisted GBL (n = 202 seventh-graders) significantly outperformed game-only conditions, with both AI groups reporting higher perceived competence (RCT; High quality). M. K. Wang et al. (2025) confirmed superior achievement through LLM-adaptive contextual games for fifth-graders. Huang and Chen (2025) found in a 2 × 2 factorial experiment (n = 160) that instructional videos produced large main effects on science learning (η^2^ = 0.27), while the AI chatbot produced significant interaction effects on extraneous cognitive load, suggesting complementary rather than redundant functions.

In language learning, Hwang and Zhang (2024) demonstrated improvements in EFL vocabulary achievement, self-efficacy, and reduced anxiety through adaptive multi-role agent GBL (n = 56). Ngu et al. (2025) found that GenAI metacognitive scaffolding significantly outperformed text-based scaffolding in terms of learning effectiveness, flow, and perceived game fidelity among workplace communication learners (n = 91). G. Tao et al. (2026) found that AI agent adaptive feedback significantly improved classroom engagement (n = 102 middle school students). However, effects on knowledge and motivation were non-significant, underscoring the importance of distinguishing outcome types.

#### 4.1.2. Higher-Order Cognitive Skills

Beyond factual recall, AI-GBL supported higher-order thinking in several included studies. As shown in Table 4, the reported effects extended beyond achievement to higher-order cognitive skills. Chernbumroong et al. (2026) demonstrated that LLM-driven NPCs aligned with the CRAAP framework produced larger gains in credibility evaluation (a critical reasoning skill) than traditional instruction. Wan et al. (2025) reported significant gains in knowledge application, critical thinking, and self-efficacy through generative AI-assisted game design as service learning. Daniels et al. (2025) found that generative AI-enhanced leadership simulations produced evidence of evolving professional identity, ethical deliberation, and collaborative confidence (n = 160; effect: +16 percentage points, t = 9.13, *p* < 0.001).

### 4.2. Motivational and Affective Dimensions

#### 4.2.1. Intrinsic Motivation and Engagement

Motivational outcomes were the most consistently positive dimension across the included studies. Damastuti et al. (2024) demonstrated that fuzzy logic + Q-learning DDA extended play sessions by 35% and improved player effectiveness by 28%. Ngu et al. (2025) found that generative AI NPC metacognitive scaffolding significantly outperformed text-based scaffolding in flow, perceived fidelity, and learning. Sun et al. (2025) documented significant motivation, confidence, and fluency gains through AI-driven gamified speech training (RCT; High quality). Gutierrez et al. (2025) reported high satisfaction ratings (4.2–4.5 out of 5) and significant correlations between RIASEC game responses and academic performance across 200 participants from high school and university settings.

#### 4.2.2. Flow, Anxiety, and Emotional Regulation

Chien et al. (2025) found high flow and low anxiety in generative AI-scaffolded role-playing games, with perceived AI usefulness significantly predicting learning assistance. M. K. Wang et al. (2025) confirmed superior flow experiences through LLM-adaptive contextual games for fifth-graders alongside reduced cognitive load. Ayyal Awwad (2025) reported a 50% reduction in negative emotional responses and 15% higher quiz scores through emotion-, gaze-, and context-driven adaptive GBL. Pituxcoosuvarn et al. (2025) found that EFL learners perceived AI word-guessing game partners as less intimidating than human partners, with modest improvements in fluency and complexity.

#### 4.2.3. Self-Efficacy and Learner Confidence

Cao et al. (2025) documented significant self-efficacy gains alongside knowledge achievement through an AI sound-tracking RPG for cultural relic restoration (n = 60). Guo et al. (2026) reported that rule-based adaptive DDA produced significantly higher creative self-efficacy (η^2^p = 0.349) than non-adaptive conditions. Daniels et al. (2025) found that generative AI-enhanced leadership simulations produced evidence of evolving professional identity, ethical deliberation, and collaborative confidence.

### 4.3. AI-Driven Personalization and Adaptive Mechanisms

#### 4.3.1. Adaptive Difficulty Adjustment

Chrysafiadi et al. (2023) demonstrated that fuzzy-logic DDA, simultaneously adjusting content and gameplay difficulty, outperformed static conditions in challenge balance. Damastuti et al. (2024) advanced this with Q-learning + fuzzy logic integration. Guo et al. (2026) showed that interpretable rule-based DDA using a performance index (accuracy + response time) produced the strongest self-efficacy and learning outcomes. Critically, Ahmed et al. (2025) found that rule-based dynamic difficulty significantly outperformed progressive difficulty in a VR shooting task (n = 50), confirming that adaptation direction, not merely presence, determines outcomes. Panjaburee et al. (2025) demonstrated in a longitudinal PLS-SEM study (n = 372) that fuzzy logic and decision tree personalisation significantly improved digital citizenship outcomes and sustained motivation over time, the largest and longest study in the included sample.

#### 4.3.2. LLM-Based and Generative AI Scaffolding

Gong et al. (2026) demonstrated that LLM scaffolding reduced cognitive load and improved achievement among fifth-graders, with higher-load students seeking AI assistance more frequently, indicating demand-responsive rather than habitual use. Huang and Chen (2025) found, in a 2 × 2 design (n = 160), that instructional videos produced main effects on science learning, while AI chatbots produced interaction effects, suggesting complementary rather than redundant functions. Fang et al. (2025) critically found that positive LLM tutor perceptions do not necessarily translate to learning gains, with content-relevant AI responses associated with more superficial engagement (the over-reliance risk). C.-H. Chen and Law (2025) confirmed that instrumental help-seeking positively predicts game performance while avoidance help-seeking predicts increased game attempts (n = 102).

#### 4.3.3. AI-Powered NPCs and Conversational Agents

Zhao et al. (2025) provided direct experimental evidence (n = 60) that AI-driven character behavior (not merely game format) drives knowledge and engagement gains in design history education. Chernbumroong et al. (2026) demonstrated gains in CRAAP-aligned LLM-NPC credibility evaluation while identifying response latency as an extraneous cognitive load. Liang et al. (2024) found that AI chatbot museum learning partners improved metacognitive awareness, emotional engagement, and double-loop learning. M. Wang et al. (2025) showed that LLM-assisted ARGs improved STEM achievement and metacognitive awareness, with low-frequency goal-directed NPC interactions outperforming high-frequency exploratory ones.

#### 4.3.4. Personalization for Diverse Populations

Aslan et al. (2025) demonstrated the feasibility of multimodal conversational AI in the Kid Space immersive physical-digital learning environment, producing 24% learning gains and high engagement metrics (physical activity 99.3% of the time; screen time reduced to 41%) among 14 first-grade students. As summarized in Table 5, AI-GBL studies also reported positive motivational and affective outcomes across diverse learning contexts. Hsu and Lin (2025) identified an aptitude-treatment interaction: experiential learning theory-based AI robot GBL benefited lower-ability students while subject-based learning benefited higher-ability students. Panjaburee et al. (2025) found that sustained motivation at later learning stages most strongly predicts the sustainability of digital citizenship behaviors over time.

### 4.4. Intelligent Assessment and Learning Analytics

#### 4.4.1. Stealth Assessment

Rahimi and Shute (2024) established the evidence-centered design framework for stealth assessment, validating psychometrically sound methods for creativity and physics understanding in Physics Playground. Gupta et al. (2024) extended this to Crystal Island (n = 119 middle school students), achieving accurate deep learning predictions of science content and reflection depth, auditing for demographic bias, and demonstrating debiasing without accuracy loss. Gupta et al. (2026) demonstrated that goal recognition significantly improved concept-level SA accuracy and enabled earlier prediction convergence beyond gameplay traces alone.

#### 4.4.2. Knowledge Tracing and Learner Modeling

Emerson et al. (2023) showed that incorporating NLP text representations of post-test assessment questions substantially improved early performance prediction in Crystal Island (n = 66 undergraduates), achieving stronger results than gameplay features alone. Goslen et al. (2025) provided data-driven analysis of explicit planning activities in Crystal Island (n = 144 middle school students), demonstrating that planning tool engagement predicted scenario completion and identifying potential for adaptive planning support. Nedombeloni et al. (2024) implemented Bayesian knowledge tracing within a telecommunications escape game, demonstrating personalised learning through statistical student modelling. These three studies represent the strongest stealth assessment evidence in the included sample, all scoring Moderate quality (17–18/25).

#### 4.4.3. Multimodal Assessment and Behavioral Analytics

Capecchi et al. (2025) demonstrated an iterative AI-NPC prompt optimization approach using zero-shot RoBERTa classification to improve alignment between NPC feedback and predefined educational objectives in a nutrition and sustainability serious game (n = 93 children), achieving significant improvements in pedagogical coverage through prompt refinement. Ma et al. (2026) employed LoRA-fine-tuned Stable Diffusion for image generation within a cultural heritage serious game, demonstrating AI-generated imagery’s capacity to foster engagement and knowledge of intangible cultural heritage.

### 4.5. Challenges, Limitations, and Emerging Directions

Cognitive load challenges include LLM latency, which introduces extraneous load (Chernbumroong et al., 2026; Mongkoljaturong et al., 2025), and the over-reliance risk identified by Fang et al. (2025) and C.-H. Chen and Law (2025), where the AI feedback format determines whether scaffolding reduces or supplants deep engagement. Ethical and fairness challenges include demographic bias in stealth assessment models (Gupta et al., 2024): of the 55 included studies, only two explicitly conducted bias audits, and one examined differential outcomes by learner ability level, indicating that equity-by-design research remains largely aspirational. Technical challenges include system latency in LLM and 3D environments (Mongkoljaturong et al., 2025) and the finding that AI presence alone does not guarantee superior outcomes: Gaurav et al. (2022) and G. Tao et al. (2026) demonstrated cases in which AI-integrated conditions did not significantly outperform conventional game-based learning on knowledge measures. Research gaps include the near absence of RCTs (n = 4, 7% of included studies), limited demographic diversity (most studies from East Asian educational systems), underdeveloped collaborative AI-GBL, and a near-total absence of longitudinal evidence beyond eight weeks.

## 5. Discussion

This systematic review synthesized 55 empirical studies to map the evidence on AI-supported game-based learning. The findings reveal a field in rapid maturation, characterized by convergent, though methodologically variable, evidence of effectiveness, an expanding repertoire of AI mechanisms, and growing sophistication in assessment methodology, alongside persistent methodological limitations and emerging ethical challenges.

### 5.1. RQ1: Effects on Learning Outcomes, Motivation, and Affect

The evidence generally supports that AI-GBL is associated with positive effects on knowledge acquisition and academic performance across diverse subject domains. However, the strength and consistency of effects vary considerably by study design, AI mechanism, and outcome type. This nuanced picture is important: across many reviewed studies, AI-GBL demonstrates positive effects relative to non-AI game-based conditions, suggesting that AI mechanisms may amplify GBL’s inherent educational value rather than merely replicating it. However, this conclusion is based on thematic synthesis of studies that vary considerably in design quality and comparison conditions; stronger causal claims await quantitative meta-analytic synthesis.

The four High-quality studies by C.-H. Chen and Chang (2024), Sun et al. (2025), Huang and Chen (2025), and Panjaburee et al. (2025), who all employed true experimental or 2 × 2 factorial designs with random or systematic group assignment, used validated outcome instruments with pre-post measurement, achieved sample sizes of at least 57, and provided transparent descriptions of their AI systems. These studies provide the strongest causal evidence for AI-GBL effectiveness in the included sample.

Motivational outcomes were generally positive. Effect sizes for motivational outcomes in AI-GBL conditions (Damastuti et al., 2024; Ngu et al., 2025) considerably exceed typical estimates for game-based learning without AI (d ≈ 0.50; Wouters et al., 2013), which is theoretically coherent with self-determination theory: AI personalization simultaneously supports competence, autonomy, and relatedness. These findings should, however, be interpreted with appropriate caution given the thematic nature of the synthesis and the substantial heterogeneity of included studies.

### 5.2. RQ2: AI Mechanisms and Adaptive Personalization

The AI mechanism landscape documented here extends prior health-domain-focused work (X. Tao et al., 2025) to the full educational context spectrum. An important finding is that interpretable, well-specified AI mechanisms tend to produce clearer learning benefits than technically sophisticated but pedagogically underspecified ones. Guo et al.’s (2026) rule-based DDA outperformed more complex Bayesian alternatives when the performance-difficulty relationship was transparent; Gupta et al.’s (2024) debiased SA model maintained accuracy while improving equity. This pattern challenges the assumption that greater algorithmic complexity necessarily produces greater educational value.

LLM scaffolding findings present a productive paradox: high learner satisfaction coexists with marginal learning gains when scaffolding enables passive reception (Fang et al., 2025). This echoes VanLehn’s (2011) finding in the intelligent tutoring literature that AI benefits depend on promoting productive struggle rather than resolving uncertainty on demand. M. Wang et al.’s (2025) finding that purposeful, low-frequency goal-directed NPC interactions outperform habitual, high-frequency exploratory ones provides actionable design guidance.

### 5.3. RQ3: Intelligent Assessment and Future Directions

The stealth assessment literature documents substantial technical progress. Prediction accuracies from Crystal Island studies (Emerson et al., 2023; Goslen et al., 2025; Gupta et al., 2024; Gupta et al. 2026) demonstrate that machine learning and deep learning approaches can provide accurate, non-intrusive assessment of learner knowledge states from gameplay logs. Gupta et al.’s (2024) explicit bias auditing, documenting gender- and experience-related disparities and demonstrating debiasing without accuracy loss, stands as best practice that the field urgently needs to generalize. Of the 55 included studies, only two explicitly examined algorithmic equity, representing a critical methodological gap as assessment models approach consequential educational decisions.

### 5.4. Theoretical and Practical Implications

Theoretically, this review contributes evidence that AI-GBL operates through three distinct pathways: cognitive load optimization (calibrating task difficulty to individual capacity; Sweller, 2011), motivational scaffolding (maintaining optimal challenge and timely feedback; Ryan & Deci, 2000), and epistemic scaffolding (AI characters supporting contextually appropriate knowledge construction; Vygotsky, 1978). This three-pathway model, supported by adaptive difficulty research (Panjaburee et al., 2025), LLM scaffolding studies (Huang & Chen, 2025; Ngu et al., 2025), and AI NPC evidence (Chernbumroong et al., 2026), explains why AI-GBL achieves outcomes that neither AI tutoring nor conventional GBL achieves alone.

For practitioners: ground adaptive mechanisms in explicit learning progression models; design AI feedback to promote active engagement rather than passive dependency; treat affective-state monitoring as a first-class design requirement; implement bias auditing as standard in any stealth assessment pipeline; and recognize AI-GBL’s equity potential for diverse and vulnerable populations. For researchers: prioritize RCT designs or factorial experiments with pre-registration; report effect sizes consistently; extend to longitudinal designs measuring knowledge retention and transfer; diversify study populations beyond East Asian secondary and university settings; and mandate bias audits for stealth assessment models.

### 5.5. Limitations

The search was conducted on a single date and restricted to two databases, potentially introducing publication bias. Despite dual-reviewer coding (κ = 0.89), the heterogeneity of AI mechanism descriptions in primary studies introduced measurement uncertainty. Thematic synthesis yields interpretive rather than quantitative effect estimates. The bespoke quality rubric has not been externally validated (a limitation explicitly acknowledged). However, its consistent application is supported by strong inter-rater reliability (κ = 0.89). The rapid pace of LLM development means that findings on generative AI mechanisms (approximately 44% of included studies) may date quickly after publication. Most included studies were conducted in East Asian educational contexts (Taiwan, China, Thailand), which substantially limited the cross-cultural generalisability of the conclusions.

## 6. Conclusions

This systematic review of 55 empirical studies provides a comprehensive synthesis of the evidence base for AI-supported game-based learning across educational contexts from 2021 to 2026. Across diverse educational settings, the reviewed studies generally indicate promising positive effects of AI-GBL through adaptive difficulty adjustment, LLM scaffolding, intelligent NPCs, and stealth assessment, with effectiveness appearing to be contingent on principled AI-instructional alignment rather than algorithmic sophistication per se. It should be noted that these conclusions are based on thematic synthesis of studies that vary considerably in design quality, outcome measures, and comparison conditions; stronger causal claims await quantitative synthesis.

The four High-quality studies in the sample (C.-H. Chen & Chang, 2024; Huang & Chen, 2025; Panjaburee et al., 2025; Sun et al., 2025) converge on a set of design principles: explicit AI-instructional alignment, transparent adaptive mechanisms, validated outcome measurement, and adequate sample sizes. These principles, rather than the specific AI technology employed, are the key determinants of meaningful learning outcomes.

Algorithmic bias in educational assessment, latency in LLM-based environments, cognitive over-reliance risks, and the near-absence of longitudinal evidence represent the field’s most pressing challenges. Equity-by-design approaches and longitudinal, demographically diverse investigations, particularly beyond East Asian educational settings, are the priority needs for the next generation of AI-GBL research.

## Figures and Tables

**Table 1:** Inclusion and Exclusion Criteria for Study Selection.

Inclusion Criteria	Exclusion Criteria
IC1. Published 2021–2026	EC1. Review/meta-analytic articles
IC2. Empirical study design	EC2. Conceptual/theoretical papers without empirical validation
IC3. AI explicitly integrated in a GBL environment	EC3. Conference papers, book chapters, or abstracts
IC4. Educational context (formal or informal)	EC4. Gamification-only studies without AI integration
IC5. At least one measurable learning-related outcome	EC5. Entertainment games without educational design intent
IC6. Peer-reviewed journal article	EC6. Studies in which AI is not involved in the learning process itself
IC7. Published in English	

Note. IC = Inclusion Criterion; EC = Exclusion Criterion.

**Table 2:** Data Extraction Coding Scheme.

Coding Category	Variables and Details Extracted
Bibliographic Information	Authors, year, journal title, country, DOI
Study Design	Research design type; sample size; participant characteristics (age, educational level)
AI Mechanism	Type of AI (LLM, GenAI, ML, RL, rule-based, Bayesian); AI role (adaptive difficulty, feedback, scaffolding, NPC, stealth assessment, learner modelling)
GBL Type	Game format (serious game, digital GBL, ARG, VR, RPG, simulation); subject domain; educational level
Learning Outcomes	Knowledge achievement, motivation, engagement, cognitive load, self-efficacy, flow, critical thinking, affective states, behavioural patterns
Assessment Instruments	Pre/post-content tests, validated questionnaires, physiological sensors, eye-tracking, behavioural logs, stealth assessment models
Key Findings & Effect Sizes	Direction of effects, magnitude, statistical significance, author-reported limitations
Quality Indicators	Research design rigor, sample size, outcome measurement, AI transparency, generalisability (per five-criterion rubric)

Note. LLM = Large Language Model; ML = Machine Learning; RL = Reinforcement Learning; NPC = Non-Player Character; GBL = Game-Based Learning.

**Table 3:** Quality Appraisal Rubric: Criteria and Scoring Anchors.

Score	Research Design	Sample Size	Outcome Measurement	AI Transparency	Generalisability	Tier
5	RCT with control group	≥200 participants	Multiple validated instruments + pre/post + follow-up	Full architecture, parameters, and code described	Multi-site or transfer/retention tested	High
4	Quasi-exp. with matched control	100–199 participants	Validated instrument + pre/post	Detailed technical description	Limitations explicitly discussed	
3	Mixed-methods or controlled comparative	50–99 participants	Pre/post (non-validated)	Partial description	Some limitation discussion	Moderate
2	Design study, survey, or case study	20–49 participants	Post-test only or single measure	General mention only	Minimal discussion	
1	Theoretical or simulation-only	<20 participants	Descriptive only	Not described	None	Low

Note. Rubric developed inductively drawing on Mayer (2019) and Wouters et al. (2013); AI Transparency criterion developed specifically for this review. Thresholds anchored to benchmarks in educational technology meta-analyses. Quality tiers: High ≥ 21 (n = 4, 7%); Moderate 16–20 (n = 31, 57%); Low ≤ 15 (n = 20, 36%). Quality appraisal used descriptively; lower-quality studies did not receive disproportionate evidential weight.

**Table 4:** Summary of Studies Reporting Learning Outcome Effects.

Citation	Domain	AI Mechanism	Effect on Achievement	Higher-Order Skills
C.-H. Chen and Chang (2024)	Science	ChatGPT GBL	Sig. gain vs. game-only (RCT, n = 202)	Active inquiry; self-monitoring
Huang and Chen (2025)	Science	GenAI Chatbot + Video	Video main effect (η^2^ = 0.27); chatbot ↓ extraneous CL	Complementary scaffold integration
Liu and Ldokova (2026)	Mathematics	GPT-Powered Games	Large sustained gains (RCT, n = 120)	Problem-solving; self-directed motivation
Sun et al. (2025)	Public Speaking	AI Speech (ASR + LLM)	Sig. motivation, confidence, fluency (RCT)	Metacognitive speech regulation
Panjaburee et al. (2025)	Digital Citizenship	Fuzzy + DT	Sig. digital citizenship gains (n = 372, longitudinal)	Ethical digital behaviour sustainability
Daniels et al. (2025)	Leadership	GenAI Simulation	+16 pp vs. control (t = 9.13, *p* < 0.001, n = 160)	Professional identity; ethical deliberation
Santos et al. (2026)	Financial Literacy	GenAI Multi-Agent	+58% earnings; −37% risky choices (n = 134)	Strategic decision-making; risk awareness
Ngu et al. (2025)	Workplace Comm.	GenAI NPC Scaffold	Higher flow, fidelity, learning vs. text (n = 91)	Metacognitive reflection
G. Tao et al. (2026)	Cybersecurity	AI Agent Feedback	Sig. classroom engagement; non-sig. knowledge	Goal engagement over content acquisition

Note. CL = Cognitive Load; RCT = Randomized Controlled Trial; ASR = Automatic Speech Recognition; DT = Decision Tree; GenAI = Generative AI; NPC = Non-Player Character; LLM = Large Language Model; pp = percentage points. The symbol (↓) indicates a reduction in extraneous cognitive load relative to the comparison condition.

**Table 5:** Summary of Motivational and Affective Outcomes in AI-GBL Studies.

Citation	Context	AI Feature	Motivational Outcome	Affective/Emotional Outcome
Damastuti et al. (2024)	Management	Fuzzy + Q-Learning DDA	+28% effectiveness; +35% session length	Reduced boredom; higher satisfaction
Ngu et al. (2025)	Workplace Comm.	GenAI NPC Scaffolding	Higher perceived fidelity; high flow	Lower anxiety; higher germane CL
Sun et al. (2025)	Public Speaking	AI Speech Recognition	Sig. motivation & confidence gains (RCT)	Reduced speaking anxiety; improved fluency
Chien et al. (2025)	Role-Play Learning	GenAI Scaffolding	High flow; low activity anxiety	Positive engagement throughout session
Ayyal Awwad (2025)	General (Children)	Emotion-/Gaze-Aware ML	Higher satisfaction (80%)	−50% negative emotions; +15% quiz scores
Gutierrez et al. (2025)	Vocational Guidance	GenAI/RIASEC	High satisfaction (4.2–4.5/5, n = 200)	Positive career exploration experience
Pituxcoosuvarn et al. (2025)	EFL Speaking	AI Word-Guess Game	Modest fluency/complexity gains	Less intimidating than human partner

Note. DDA = Dynamic Difficulty Adjustment; GenAI = Generative AI; NPC = Non-Player Character; CL = Cognitive Load; RCT = Randomized Controlled Trial; ML = Machine Learning.

## Data Availability

The data supporting the findings of this study are contained within the article. No new data were created or analyzed beyond the published records included in the systematic review.

## Supplementary Materials

The following supporting information can be downloaded at: https://www.mdpi.com/article/10.3390/bs16071050/s1, PRISMA 2020 Checklist.

## Appendix A

**Table A1:** Characteristics of All Included Studies (n = 55).

No	Citation	Subject Area	AI Mechanism	Study Design	Participants	Key Finding	IC
1	Kulkarni et al. (2025)	Mathematics	Minimax AI (depth-limited rule-based)	Mixed-Methods	Primary school	AI math game improved arithmetic across three school types; teacher satisfaction high	IC1–IC7 ✓
2	Chernbumroong et al. (2026)	Digital Literacy/Misinformation	LLM-NPC (GenAI, CRAAP framework)	Mixed-Methods	Undergraduates	LLM NPCs improved misinformation detection and digital literacy skills	IC1–IC7 ✓
3	Xiao et al. (2025a)	EFL Writing	GenAI-NPC (Poe/GPT-3.5-Turbo, prompt engineering)	Pilot Study	EFL undergraduates	Positive student perceptions; situational interest and autonomy high; very limited generalizability	IC1–IC7 ✓
4	Xiao et al. (2025b)	EFL Writing	ChatGPT-NPC (storyline-based, prompt-engineered)	Quasi-Experimental	EFL undergraduates (2 classes)	ChatGPT NPCs enhanced affective and cognitive writing outcomes vs. scripted-NPC control	IC1–IC7 ✓
5	Zhao et al. (2025)	Design History	AI Virtual Characters (adaptive guidance, role-playing)	Quasi-Experimental	College students	AI characters significantly improved engagement and knowledge vs. non-AI game control	IC1–IC7 ✓
6	Y. Chen and Hou (2024)	Workplace Ethics	ChatGPT-NPC Scaffolding (RAG framework)	Quasi-Experimental	University students	ChatGPT NPC scaffolding enhanced ethics learning, motivation, and engagement	IC1–IC7 ✓
7	Ngu et al. (2025)	Workplace Communication	GenAI-NPC Metacognitive Scaffolding (Gather Town, IRB)	Quasi-Experimental	University students (adults)	GenAI scaffolding outperformed video and text on learning effectiveness, flow, and game fidelity	IC1–IC7 ✓
8	Gong et al. (2026)	AI Literacy	LLM-Based Scaffolding (fifth-grade, quasi-exp)	Quasi-Experimental	Fifth-grade students	LLM scaffolding significantly improved AI literacy and cognitive engagement vs. control	IC1–IC7 ✓
9	Chien et al. (2025)	AI Prompting Skills	GenAI Scaffolding (ChatPDF + ChatGPT API)	Single-Group Observational	University students	High flow and low anxiety reported; 83%+ accuracy in chatbot responses; no comparison group	IC1–IC7 ✓
10	C.-H. Chen and Chang (2024)	Science	ChatGPT-Assisted GBL (with vs. without few-shot examples)	RC	7th-grade students	ChatGPT-with-examples group significantly outperformed controls on science achievement and motivation	IC1–IC7 ✓
11	Chu et al. (2023)	EFL Vocabulary	Expert System SRL Scaffolding (rule-based, 12 IF-THEN rules)	Quasi-Experimenta	College EFL students	SRL-guided game improved vocabulary achievement and self-regulation vs. game-only group (d = 1.09)	IC1–IC7 ✓
12	Hwang and Zhang (2024)	EFL/Language	Adaptive Agents (Decision Tree, 3 roles: teacher/peer/student)	Quasi-Experimental	Junior high school students	Adaptive multi-role agents improved language achievement and self-efficacy and reduced anxiety vs. fixed-role	IC1–IC7 ✓
13	G. Tao et al. (2026)	Cybersecurity/Internet Principles	AI Agent Adaptive Feedback (RPG platform)	Quasi-Experimental	Middle school students	AI agent significantly improved classroom engagement; moderate, non-significant effects on knowledge/motivation	IC1–IC7 ✓
14	Cao et al. (2025)	Cultural Relic Restoration	AI Sound-Tracking RPG (audio-adaptive AI)	Quasi-Experimental	Undergraduate students	ST-AI RPG significantly improved knowledge achievement and self-efficacy vs. traditional RPG	IC1–IC7 ✓
15	Sun et al. (2025)	Public Speaking	AI Speech Framework (ASR + Sentiment Analysis + LLM)	True Experimental/RCT	Primary school students	AI-GSLF significantly outperformed control on all speech metrics, motivation, and immersion	IC1–IC7 ✓
16	Liang et al. (2024)	Science/Museum Learning	AI Chatbot Learning Partner (ARG context)	Quasi-Experimental	University students	AI chatbot improved self-regulated learning and metacognitive awareness in alternate reality game	IC1–IC7 ✓
17	M. Wang et al. (2025)	STEM	LLM-Agent ARG (adaptive, metacognitive scaffolding)	Quasi-Experimental	Secondary students	LLM-ARG system significantly enhanced academic performance, metacognition, and STEM engagement	IC1–IC7 ✓
18	M. K. Wang et al. (2025)	Science	LLM Adaptive Contextual Game (LLM-ACG, lag sequential analysis)	Quasi-Experimental	Fifth-grade students	LLM-ACG improved science knowledge and flow; reduced cognitive load; promoted positive learning behaviors	IC1–IC7 ✓
19	Guo et al. (2026)	Art/Human Anatomy	Rule-Based Adaptive DDA (performance index, ADDIE model)	Quasi-Experimental	First-year university students	Adaptive difficulty improved learning achievement and creative self-efficacy vs. fixed difficulty	IC1–IC7 ✓
20	Mongkoljaturong et al. (2025)	Job Interview Skills	LLM + MetaHuman AI Interviewer (wav2vec2, ChatGPT 3.5)	Pilot Study	University graduates	LLM interview simulator showed high usability; no pre-post learning comparison conducted	IC1–IC7 ✓
21	Hare et al. (2024)	Digital Logic/Engineering	ITS + RL-Based Adaptive GBL (PING, 3-year longitudinal)	Longitudinal Quasi-Experimental	Engineering undergraduates	Adapted PING system significantly improved problem-solving outcomes vs. non-adapted (d = 1.15)	IC1–IC7 ✓
22	Wan et al. (2025)	Financial Literacy/Service-Learning	GenAI-Assisted Game Design (S-LOMS, sequential explanatory)	Mixed-Methods	University students	GenAI-supported group showed higher knowledge application, critical thinking, and self-efficacy	IC1–IC7 ✓
23	Huang and Chen (2025)	Science (Physics)	GenAI Chatbot (ChatGPT 3.5-Turbo) + Instructional Videos (2 × 2 factorial)	True Experimental/RCT	7th-grade students	Instructional videos (eta^2^ = 0.27) improved science learning; GenAI reduced extraneous cognitive load	IC1–IC7 ✓
24	Liu and Ldokova (2026)	Mathematics	GPT-Powered Adaptive Video Games (RCT)	RCT	Adolescents	GPT-powered games significantly improved math achievement and motivation vs. control	IC1–IC7 ✓
25	Santos et al. (2026)	Financial Literacy	GenAI Multi-Agent (QD-based, 3-phase adaptive architecture)	Empirical Case Study	Children	AI personalization shaped learning trajectories across behavioral, cognitive, and motivational dimensions	IC1–IC7 ✓
26	Parrales-Bravo et al. (2026)	Health Literacy (Cancer Awareness)	AI-Generated Quiz Feedback (CONGA system)	Quasi-Experimental	Engineering university students	AI-assisted game improved colon cancer literacy and critical health reflection	IC1–IC7 ✓
27	Panjaburee et al. (2025)	Digital Citizenship/Ethics	Fuzzy Logic + Decision Tree Personalized GBL (PLS-SEM)	Longitudinal Repeated-Measures	Lower secondary students (Thailand)	Personalized GBL significantly improved digital citizenship; PLS-SEM modeled sustainability perspectives	IC1–IC7 ✓
28	Fang et al. (2025)	Chinese as a Foreign Language	LLM Tutor (iFlytek Spark LLM, dual-language output)	Single-Group Observational	Beginner CFL learners	LLM tutor well-received (high PE); actual learning gains limited; off-topic responses fostered deeper cognition	IC1–IC7 ✓
29	Ayyal Awwad (2025)	General/Mobile Learning	ML Emotion + Eye-Gaze Adaptation (Pocket Code, DDA)	Pilot Study	Children (8–13 years)	Adaptive GBL reduced negative emotions by 50%; improved quiz scores; very small sample	IC1–IC7 ✓
30	Pituxcoosuvarn et al. (2025)	EFL Speaking	AI Partner (Taboo Talks word-guessing, CAF metrics)	Counterbalanced Quasi-Experimental	EFL learners	AI partner improved fluency and complexity; learners perceived AI partner as less intimidating than humans	IC1–IC7 ✓
31	Emerson et al. (2023)	Science (Microbiology)	ML Stealth Assessment (BKT + Deep Learning, Crystal Island)	Empirical Behavioral Analysis	Undergraduates	Incorporating post-test embeddings improved early knowledge prediction accuracy in GBL	IC1–IC7 ✓
32	Goslen et al. (2025)	Science (Microbiology)	ML Plan Recognition + Adaptive Scaffolding (Crystal Island)	Empirical Behavioral Analysis	Middle school students	Planning tool engagement predicted scenario completion; data-driven SRL pattern analysis presented	IC1–IC7 ✓
33	Gupta et al. (2024)	Science (Microbiology)	Deep Learning Stealth Assessment (NLP + reflection scoring)	Empirical Behavioral Analysis	Middle school students	DL-SA predicted reflection depth and post-test scores; bias analysis across gender conducted	IC1–IC7 ✓
34	Gupta et al. (2026)	Science (Microbiology)	Deep Learning + Goal Recognition SA (Crystal Island)	Empirical Behavioral Analysis	Middle school students	Goal recognition enhanced concept-level SA accuracy beyond gameplay traces alone	IC1–IC7 ✓
35	Dai et al. (2023)	Mathematics	AI Support Tools (Task Planner + Math Storyboard)	Mixed-Methods	Elementary students	AI tools influenced math learning behaviors; mixed scaffolding patterns across task types	IC1–IC7 ✓
36	Nedombeloni et al. (2024)	Telecommunications/CS	Bayesian Knowledge Tracing (BKT, escape room serious game)	Empirical Design Study	University students	BKT-personalized escape room improved personalization vs. streak-based assessment approach	IC1–IC7 ✓
37	Chrysafiadi et al. (2023)	Computer Science (HTML)	Fuzzy Logic DDA (3D educational game)	Quasi-Experimental	CS students	Fuzzy DDA improved motivation and engagement in HTML programming game vs. static difficulty	IC1–IC7 ✓
38	Wenzel et al. (2026)	Entrepreneurship/Business	AI Conversational Agent (Adaptive Feedback, BSG)	Quasi-Experimental	Business students	AI conversational agent improved formative feedback quality and learning outcomes in business simulation	IC1–IC7 ✓
39	Gaurav et al. (2022)	Cybersecurity	ML-Adaptive Agent (Classification-based, SQL/firewall)	Quasi-Experimental	CS students	ML-adaptive game marginally improved cybersecurity learning vs. non-adaptive baseline	IC1–IC7 ✓
40	Damastuti et al. (2024)	Management/VR Gaming	Q-Learning + Fuzzy Logic DDA (Unreal Engine)	Controlled Player Study	Game players	Q-learning + fuzzy DDA increased play duration 35% and player satisfaction vs. static DDA	IC1–IC7 ✓
41	Aster et al. (2024)	Medical Education	AI Chatbot + Virtual Patient Generator (AI-generated faces)	Usability Study	Undergraduate medical students	Emergency department serious game showed substantial usability (SUS); learning outcomes not yet measured	IC1–IC7 ✓
42	Gutierrez et al. (2025)	Vocational Guidance	AI Generative Scenarios (RIASEC + OpenAI, real-time)	Mixed-Methods	High school and university students	High satisfaction (4.2–4.5/5) and significant correlation between RIASEC game responses and academic performance	IC1–IC7 ✓
43	C.-H. Chen and Law (2025)	Physics	ChatGPT Help-Seeking (Summon of Magicrystal GBL)	Empirical Behavioral Analysis	Middle school students	Instrumental help-seeking positively predicted game performance; avoidance predicted game attempts	IC1–IC7 ✓
44	Aslan et al. (2025)	Collaborative Problem Solving (Early Childhood)	Multimodal Conversational AI (Kid Space, wall projection)	Quasi-Experimental + Case Study	Elementary students + 3 educators	Kid Space produced 24% learning gains; high engagement with reduced screen time (41%)	IC1–IC7 ✓
45	Maskeliūnas et al. (2023)	Programming Education	Pareto Optimization + Gamification Framework (PWA, FGPE+)	Multi-Site Empirical	Programming students (Poland + Lithuania)	Pareto-optimized FGPE+ outperformed Moodle on perceived knowledge; positive UEQ across both countries	IC1–IC7 ✓
46	Shum et al. (2025)	Programming Education	Fuzzy Logic DDA (MSBD + ARCS framework, GhostCoder)	Single-Group Pre-Post	Vocational IT students	MSBD framework significantly improved programming knowledge (t = 3.216, *p* < 0.05); high IMMS motivation scores	IC1–IC7 ✓
47	Ulrich et al. (2024)	Italian Sign Language (LIS)	ML Gesture Recognition (MediaPipe + Random Forest, 99.83% accuracy)	Usability Study	Primary school children	SIGNIFY achieved near-perfect gesture recognition; positive child usability feedback; limited generalizability	IC1–IC7 ✓
48	Ahmed et al. (2025)	Shooting Skills/VR	Dynamic Difficulty Adjustment (Rule-based AI, VR hypercasual game)	Quasi-Experimental	Adult VR learners	Dynamic difficulty (DGD) significantly outperformed progressive difficulty (PGD) on shooting performance	IC1–IC7 ✓
49	Hsu and Lin (2025)	Computational Thinking/AI	AI Image Recognition + Robot GBL (ELT vs. SBL)	Quasi-Experimental	Undergraduates	Both ELT and SBL improved CT and AI knowledge; ELT more effective for lower-ability learners (ANCOVA)	IC1–IC7 ✓
50	Capecchi et al. (2025)	Nutrition/Sustainability	AI-NPC (Zero-shot RoBERTa, iterative prompt optimization)	Iterative Design Study	Children (Generation Alpha)	Prompt refinement significantly improved NPC coverage of nutritional and sustainability learning objectives	IC1–IC7 ✓
51	Ma et al. (2026)	Intangible Cultural Heritage	AI Drawing (LoRA fine-tuned Stable Diffusion, Img2Img)	Empirical Mixed-Methods	Cultural heritage learners	AI-generated imagery in DongSeasons fostered ICH engagement; experimental group outperformed control	IC1–IC7 ✓
52	Sunday et al. (2023)	Object-Oriented Programming	AI Error Feedback Agent (Imikode VR, intelligent agent)	Usability Study (n = 103)	First-year university CS students	Imikode VR achieved 90%+ acceptance on USE questionnaire; AI feedback perceived as beneficial	IC1–IC7 ✓
53	Aslan et al. (2022)	Early Childhood Math	Multimodal AI (Kid Space Pilot, wizard-in-loop, n = 8)	Exploratory Case Study	First-grade students	High on-task behavior (99.2%); positive emotional engagement; strong novelty effect; very limited sample	IC1–IC7 ✓
54	Aslan et al. (2024)	Early Childhood Math	Conversational AI Agent (Kid Space, full deployment)	Quasi-Experimental + Case Study	Elementary students + 3 educators	Engagement metrics improved (screen time 41%, physical activity 99.3%, social interaction 52%)	IC1–IC7 ✓
55	Daniels et al. (2025)	Project Management/Leadership	GenAI Simulation (Adaptive Decision-making, VLE-embedded)	Mixed-Methods	Undergraduate and postgraduate students	AI-GBL group outperformed traditional case-based control by 16 percentage points (t = 9.13, *p* < 0.001)	IC1–IC7 ✓

Note. IC1 = Published 2021–2026; IC2 = Empirical study; IC3 = AI integrated in GBL; IC4 = Educational context; IC5 = Measurable learning outcome; IC6 = Peer-reviewed journal; IC7 = English language. Key abbreviations: LLM = Large Language Model; GenAI = Generative AI; NPC = Non-Player Character; DDA = Dynamic Difficulty Adjustment; SA = Stealth Assessment; BKT = Bayesian Knowledge Tracing; ITS = Intelligent Tutoring System; SRL = Self-Regulated Learning; EFL = English as a Foreign Language; CFL = Chinese as Foreign Language; RCT = Randomized Controlled Trial; ARG = Alternate Reality Game; ML = Machine Learning; VR = Virtual Reality; ASR = Automatic Speech Recognition; PWA = Progressive Web Application; RPG = Role-Playing Game; QD = Quality-Diversity; ICH = Intangible Cultural Heritage; RIASEC = Realistic Investigative Artistic Social Enterprising Conventional; BSG = Business Simulation Game; CAF = Complexity Accuracy Fluency; USE = Usefulness Satisfaction Ease of Use; IMMS = Instructional Materials Motivation Survey; UEQ = User Experience Questionnaire; SUS = System Usability Scale; PLS-SEM = Partial Least Squares SEM; CRAAP = Currency Relevance Authority Accuracy Purpose; LIS = Italian Sign Language. ✓ indicates that the study met the corresponding inclusion criterion (IC).

## Appendix B

**Table A2:** Methodological Quality Assessment of Included Studies (n = 55).

No	Citation	Year	D1	D2	D3	D4	D5	Total	Quality Tier
1	Kulkarni et al. (2025)	2025	3	4	4	5	3	19	Moderate
2	Chernbumroong et al. (2026)	2026	4	3	4	4	3	18	Moderate
3	Xiao et al. (2025a)	2025	2	1	3	3	1	10	Low
4	Xiao et al. (2025b)	2025	3	3	4	4	3	17	Moderate
5	Zhao et al. (2025)	2025	3	2	3	3	2	13	Low
6	Y. Chen and Hou (2024)	2024	3	2	3	4	2	14	Low
7	Ngu et al. (2025)	2025	4	3	5	4	3	19	Moderate
8	Gong et al. (2026)	2026	3	3	4	4	3	17	Moderate
9	Chien et al. (2025)	2025	2	3	5	4	2	16	Moderate
10	C.-H. Chen and Chang (2024)	2024	5	4	4	4	4	21	High
11	Chu et al. (2023)	2023	3	2	4	4	2	15	Low
12	Hwang and Zhang (2024)	2024	3	3	5	4	3	18	Moderate
13	G. Tao et al. (2026)	2026	3	4	4	4	3	18	Moderate
14	Cao et al. (2025)	2025	3	2	4	3	3	15	Low
15	Sun et al. (2025)	2025	5	3	5	5	3	21	High
16	Liang et al. (2024)	2024	3	3	4	3	3	16	Moderate
17	M. Wang et al. (2025)	2025	3	3	4	4	3	17	Moderate
18	M. K. Wang et al. (2025)	2025	3	3	4	4	3	17	Moderate
19	Guo et al. (2026)	2026	3	4	4	4	3	18	Moderate
20	Mongkoljaturong et al. (2025)	2025	2	1	2	5	1	11	Low
21	Hare et al. (2024)	2024	4	4	4	4	3	19	Moderate
22	Wan et al. (2025)	2025	4	3	4	3	3	17	Moderate
23	Huang and Chen (2025)	2025	5	4	5	4	3	21	High
24	Liu and Ldokova (2026)	2026	5	3	4	4	3	19	Moderate
25	Santos et al. (2026)	2026	2	3	3	5	2	15	Low
26	Parrales-Bravo et al. (2026)	2026	3	4	3	3	3	16	Moderate
27	Panjaburee et al. (2025)	2025	4	5	4	4	4	21	High
28	Fang et al. (2025)	2025	2	2	3	4	2	13	Low
29	Ayyal Awwad (2025)	2025	2	1	2	3	1	9	Low
30	Pituxcoosuvarn et al. (2025)	2025	4	2	4	3	2	15	Low
31	Emerson et al. (2023)	2023	3	3	4	4	3	17	Moderate
32	Goslen et al. (2025)	2025	3	3	4	4	3	17	Moderate
33	Gupta et al. (2024)	2024	3	3	4	5	3	18	Moderate
34	Gupta et al. (2026)	2026	3	3	4	5	3	18	Moderate
35	Dai et al. (2023)	2023	4	3	4	3	3	17	Moderate
36	Nedombeloni et al. (2024)	2024	3	2	4	4	2	15	Low
37	Chrysafiadi et al. (2023)	2023	3	2	3	4	2	14	Low
38	Wenzel et al. (2026)	2026	3	3	4	4	3	17	Moderate
39	Gaurav et al. (2022)	2022	3	3	3	3	3	15	Low
40	Damastuti et al. (2024)	2024	3	2	3	5	2	15	Low
41	Aster et al. (2024)	2024	2	3	3	4	2	14	Low
42	Gutierrez et al. (2025)	2025	4	4	4	4	4	20	Moderate
43	C.-H. Chen and Law (2025)	2025	3	3	4	4	3	17	Moderate
44	Aslan et al. (2025)	2025	4	2	4	4	2	16	Moderate
45	Maskeliūnas et al. (2023)	2023	3	3	3	4	3	16	Moderate
46	Shum et al. (2025)	2025	2	2	3	4	2	13	Low
47	Ulrich et al. (2024)	2024	2	1	2	4	1	10	Low
48	Ahmed et al. (2025)	2025	3	2	4	3	2	14	Low
49	Hsu and Lin (2025)	2025	3	3	4	4	3	17	Moderate
50	Capecchi et al. (2025)	2025	2	3	4	5	2	16	Moderate
51	Ma et al. (2026)	2026	4	2	5	5	2	18	Moderate
52	Sunday et al. (2023)	2023	2	4	3	2	2	13	Low
53	Aslan et al. (2022)	2022	2	1	3	3	1	10	Low
54	Aslan et al. (2024)	2024	4	2	4	4	2	16	Moderate
55	Daniels et al. (2025)	2025	4	4	4	4	3	19	Moderate

Note. Quality scores assigned after full-text review (where available) supplemented by structured abstract analysis. D1 = Research Design (1 = descriptive/case study; 2 = single-group pre-post; 3 = quasi-experimental; 4 = longitudinal/multi-group; 5 = RCT/true experimental). D2 = Sample Size (1 = <20; 2 = 20–49; 3 = 50–99; 4 = 100–199; 5 = ≥200). D3 = Outcome Measurement Validity (1 = usability/engagement only; 2 = single unvalidated measure; 3 = validated instrument or pre-post-test; 4 = multiple validated measures; 5 = validated instruments + pre-post + objective measure). D4 = AI Transparency (1 = not described; 2 = briefly mentioned; 3 = partially described; 4 = moderately described with some technical detail; 5 = fully described with algorithm/model specifications). D5 = Generalizability (1 = <20 participants, single session; 2 = small N single context; 3 = moderate N or multiple groups; 4 = large N or multi-context; 5 = multi-site, large N, longitudinal). Quality tiers: High ≥21 (n = 4, 7%); Moderate 16–20 (n = 31, 57%); Low ≤15 (n = 20, 36%). Mean score = 16.1 (SD = 2.9, range = 9–21).
